# Possible Rehabilitation Procedures to Treat Sarcopenic Dysphagia

**DOI:** 10.3390/nu14040778

**Published:** 2022-02-12

**Authors:** Hitoshi Kagaya, Yoko Inamoto

**Affiliations:** 1 Department of Rehabilitation Medicine I, School of Medicine, Fujita Health University, Toyoake 470-1192, Japan; 2 Faculty of Rehabilitation, School of Health Sciences, Fujita Health University, Toyoake 470-1192, Japan; inamoto@fujita-hu.ac.jp

**Keywords:** dysphagia, sarcopenic dysphagia, resistance training, rehabilitation procedure

## Abstract

Sarcopenic dysphagia requires the presence of both dysphagia and generalized sarcopenia. The causes of dysphagia, except for sarcopenia, are excluded. The treatment for sarcopenic dysphagia includes resistance training along with nutritional support; however, whether rehabilitation procedures are useful remains unclear. In this narrative review, we present possible rehabilitation procedures as a resistance training for managing sarcopenic dysphagia, including Shaker exercise, Mendelsohn maneuver, tongue-hold swallow exercise, jaw-opening exercise, swallow resistance exercise, lingual exercise, expiratory muscle strength training, neuromuscular electrical stimulation, and repetitive peripheral magnetic stimulation. We hope that some procedures mentioned in this article or new methods will be effective to treat sarcopenic dysphagia.

## 1. Introduction

The term sarcopenia was advocated in 1989 [1]. Sarcopenia is defined as a progressive and generalized skeletal muscle disorder associated with an increased likelihood of adverse outcomes, including falls, fractures, physical disability, and mortality according to the European Working Group on Sarcopenia in Older People (EWGSOP2). Probable sarcopenia is identified by low muscle strength [2]. Similarly, the Asian Working Group for Sarcopenia (AWGS) adopted the definition for sarcopenia as a decrease in skeletal muscle mass and muscle strength or physical function in elderly individuals [3,4]. The reported prevalence rates of sarcopenia were 1–29% in community-dwelling populations, 14–33% in long-term care populations, and 10% in the only acute hospital-care population examined [5].

**Figure 1:**
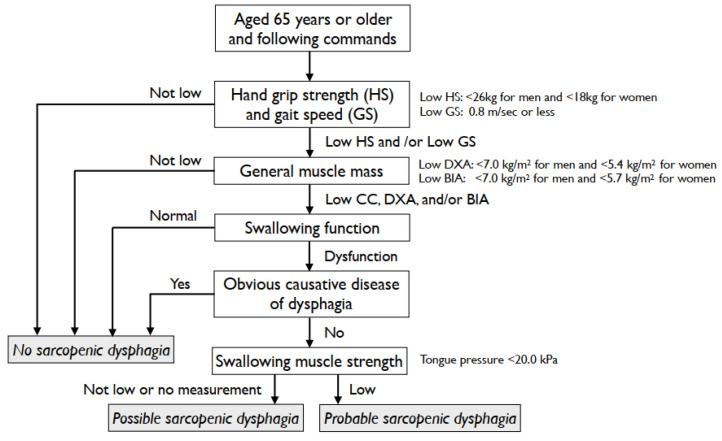
Diagnostic algorithm for sarcopenic dysphagia [11,12]. CC, calf circumference; DXA, dual-energy X-ray absorptiometry; BIA, bioimpedance analysis.

Malnutrition is a major factor contributing to increased morbidity and mortality, decreased function and quality of life, increased frequency and length of hospital stay, and higher healthcare costs [6]. It is associated with an approximately fourfold higher risk of developing sarcopenia [7]. In addition, sarcopenic obesity is a significant predictor of all-cause mortality among older people, particularly in hospitalized patients [8]. Even for sarcopenic obesity, current treatment strategies are calorie restriction, protein supplementation, and aerobic and resistance exercises [9]. Therefore, enteral nutrition, such as nasogastric tube feeding, percutaneous endoscopic gastrostomy (PEG), or percutaneous endoscopic jejunostomy (PEJ), is used to prevent malnutrition for patients with dysphagia who have difficulty in eating and drinking. Dysphagia is common in stroke, chronic obstructive pulmonary disease, and neuromuscular diseases, among others. In addition, the term “sarcopenic dysphagia” was first used in 2012 [10]. The advocating diagnostic criteria are the presence of dysphagia and generalized sarcopenia. The causes of dysphagia, except for sarcopenia, are excluded. Figure 1 shows the diagnostic algorithm for sarcopenic dysphagia [11,12]. The odds ratio for the association between whole-body sarcopenia and dysphagia was 4.06 [13]. The patients with probable sarcopenic dysphagia showed more severe malnutrition than those with possible sarcopenic dysphagia [14]. Aggressive nutrition management is a key issue to treat sarcopenic dysphagia. The daily energy expenditure, accumulation, and requirement should be calculated to increase the body weight and muscle mass of the patients [15]. The tongue muscle mass in patients with sarcopenic dysphagia was smaller than that in those without sarcopenic dysphagia. In addition, sarcopenic dysphagia is associated with increased tongue muscle intensity [16].

The swallowing muscles are considered striated muscles; however, their embryological characteristics are different from those of somatic muscles, which compose the skeletal muscles of the extremities [12]. The suprahyoid muscles, which consisted of the geniohyoid, mylohyoid, digastric, and stylohyoid muscles, raise the hyoid bone during swallowing. Sarcopenia of these muscles may play an important role in reducing hyoid bone movement during aging and the subsequent increased risk of aspiration in older persons. The decreased cross-sectional area of the geniohyoid muscles has been associated with aging and is greater in aspirators than that in non-aspirators [17]. Furthermore, the cross-sectional area of the geniohyoid muscles correlated with tongue pressure and jaw-opening strength [18]. To date, however, only few studies have reported the treatment of sarcopenic dysphagia.

In four case reports, nutritional support, physical exercise, and dysphagia rehabilitation were provided to patients with sarcopenic dysphagia [19,20,21,22]. Dysphagia rehabilitation included resistance training of the swallowing muscles, lingual resistance exercises, and/or head-lift exercises. Their body mass index increased from 9.4–15.3 to 14.1–18.2 kg/m^2^, and the activities of daily living improved. Initially, all cases had no oral intake; however, at the final follow up, they could eat using their mouths. A nutritional support and home-based rehabilitation, including neck muscle training, improved the swallowing function and nutrition status in a patient with Parkinson’s disease [23]. Alternatively, one randomized controlled study has examined the effects of resistance training on the swallowing muscles in community-dwelling older individuals with dysphagia. The anterior tongue was positioned longitudinally along the hard palate, posterior to the alveolar ridge, and the participants pressed the anterior aspect of their tongues against the hard palate as forcefully as they could during the tongue resistance exercise, while the hand/palm pressure to the forehead as an attempt was made by the participant to flex their head as forcefully as possible so that they could visualize their umbilicus in the head flexion exercise. Both exercises were performed for 10 s each; one set equaled to 10 attempts/repetitions. Two sets of these exercises were instructed to perform per day, three times per week, for 3 months. However, nearly half of the participants had performed resistance training less than twice weekly in reality, and these exercises failed to improve dysphagia or tongue pressure [24]. Exercise intensity may be inadequate because once or twice weekly training of major muscle groups at a moderate intensity is required to improve sarcopenia [25].

The treatment for sarcopenic dysphagia requires both resistance training of the swallowing muscles and nutritional support [12]. The resistance exercise refers to exercise performed with any type of resistance against muscles. The recent scoping review mainly focused on diagnosis and nutrition support for sarcopenic dysphagia. No details of rehabilitation procedures were described [12,15]. Therefore, this narrative review describes the possible rehabilitation procedures as resistance exercises to improve sarcopenic dysphagia.

## 2. Resistance Exercises for Dysphagia

Many procedures have been postulated for dysphagia rehabilitation. Shaker exercise [26,27], Mendelsohn maneuver [28,29], super-supraglottic swallow [30], thermal-tactile stimulation [30], tongue-hold swallow exercise [31], expiratory muscle strengthening (EMST) [32], balloon catheter dilation [33,34], neuromuscular electrical stimulation (NMES) [35,36], transcranial direct current stimulation (tDCS) [37,38], and repetitive transcranial magnetic stimulation (rTMS) [39,40] are examples. Among these procedures, some are classified as a resistance exercise, but others are not. In addition, postural adjustments, such as head turn [30] or reclining position [41,42], and dietary modifications belong to compensatory approaches. These interventions contribute to safe swallowing and may improve nutritional status by increasing oral intake. Because resistance exercises are needed to treat sarcopenic dysphagia [12], the following exercises may be effective for managing it. These exercises can be applied irrespective of the severity of dysphagia if the patients follow the instructions since they do not use food.

### 2.1. Shaker Exercise and Related Exercises

**Figure 2:**
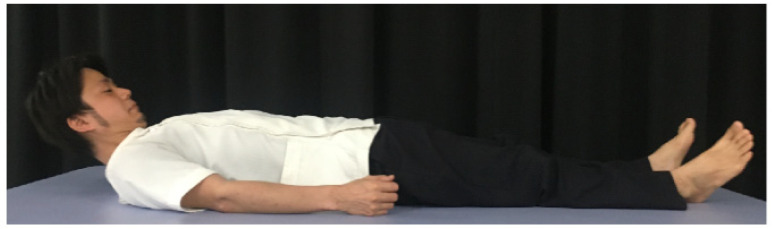
Shaker exercise. The head is kept raised in the supine position.

The Shaker exercise [26,27], one of a head-lift exercises, is a widely used rehabilitation procedure. The Shaker exercise consists of two exercises performed in the supine position. The first exercise is an isometric exercise in which the head is kept raised for 1 min and is repeated thrice at 1 min intervals (Figure 2). The second exercise is an isotonic exercise in which the head is repeatedly raised 30 times. Three sets are performed daily for 6 weeks. This exercise strengthens the suprahyoid muscles and improves hyoid laryngeal elevation. Furthermore, it increases the opening of the upper esophageal sphincter (UES) [27,43]. The effects of this exercise on patients with dysphagia were proven using a meta-analysis and a systematic review [44,45]. However, in the Shaker exercise, completing the protocol is difficult because of a significant exercise burden, often resulting in dropouts [43,46]. Moreover, in a study using surface electromyography (EMG), fatigue occurred faster in the sternocleidomastoid muscle than in the suprahyoid muscle group, affecting the quality and efficacy of the training [47,48].

Several modified Shaker exercises were developed. The most simple modification is reducing the frequency of the Shaker exercise to once a day, but it is proven to be still effective [49]. To reduce exercise intensity, the Shaker exercise performed at a 45° reclining position was postulated as a recline exercise [50]. In a randomized clinical trial, this exercise produced similar gains and de-training effects on healthy elderly adults with less effort [51]. The chin tuck against resistance (CTAR) exercise uses an inflatable 12 cm rubber ball. The patients are instructed to seat upright on a chair and hold the rubber ball between the base of the chin and the manubrium sterni. This exercise consists of isometric and isotonic movements. The isometric task was performed for 10 s, whereas the isokinetic task was successively repeated 10 times for strengthening the suprahyoid and infrahyoid muscles. Moreover, the CTAR exercise increases the activity of the suprahyoid muscles compared with the Shaker exercise [52,53].

### 2.2. Mendelsohn Maneuver (MM)

The MM is a voluntary prolongation of hyolaryngeal elevation at the peak of swallowing [28]. The MM increased the duration of the anterosuperior excursion of the larynx and hyoid and consequently delayed sphincter closure by maintaining traction on the anterior sphincter wall [29]. Therefore, the MM enhances opening of the UES by a volitional increase in the duration of submental muscle contraction during swallowing. The MM has been widely used as a compensatory strategy to improve UES opening for patients who have insufficient UES opening because of decreased laryngeal elevation. The MM not only increases the duration of UES opening, but also alters the pressure distribution during pharyngeal swallowing. The hyoid bone was positioned significantly higher at maximum displacement. Changes in the timing and magnitude of hyoid displacements and prolonged closure of the pharynx occur during swallowing [54]. The MM is somewhat difficult to perform; thus, the use of biofeedback by surface EMG seems to be effective [55].

### 2.3. Tongue-Hold Swallow Exercise

Contact between the tongue base and pharyngeal wall during swallowing is an important component in the generation of the bolus, driving pressure during the oropharyngeal phase of swallowing. Tongue-hold swallowing, also known as the Masako maneuver, is an exercise of dry swallowing while holding the anterior portion of the tongue between the upper and lower teeth, which potentially increases posterior pharyngeal wall motion during swallowing [31,56,57,58]. Recently, an increase in tongue pressure and submental surface EMG activities during tongue-hold swallowing were reported, indicating a strengthening of the tongue and suprahyoid muscles as additional possible effects of tongue-hold swallowing [59,60]. The intensity of exercise is recommended to adjust by tongue protrusion length, which is determined by the maximum tongue protrusion length [59,60]. However, so far, only one case study has reported the exercise effect of tongue-hold swallowing, and thus, the frequency and intensity of the exercise have not been sufficiently elucidated. Three months of tongue-hold swallowing only increased the contact between the tongue base and posterior pharyngeal wall in one patient with dysphagia [61].

### 2.4. Jaw-Opening Exercise

**Figure 3:**
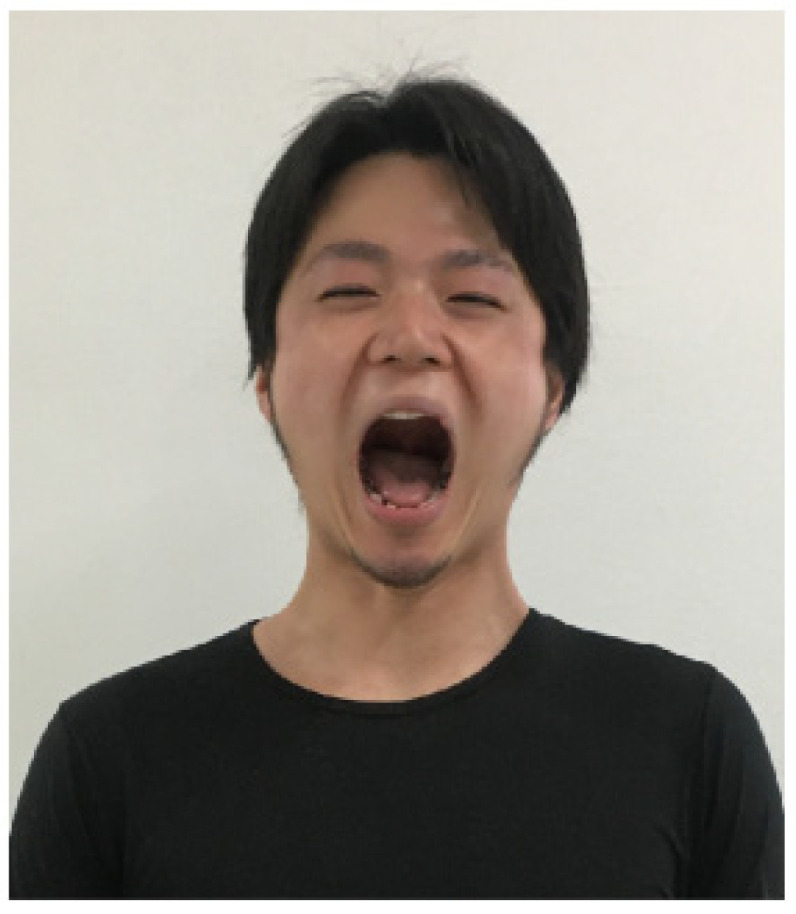
Jaw-opening exercise. The subjects opened their jaws to the maximum extent.

The mylohyoid muscle, the anterior belly of the digastric muscles, and the geniohyoid muscle are activated not only during hyoid elevation, but also during jaw opening. Jaw-opening exercises were developed to strengthen the suprahyoid muscles. The subjects opened their jaws to the maximum extent and maintained this position for 10 s five times with a 10 s rest (Figure 3). The subjects were instructed to perform two sets of this exercise daily for 4 weeks. Significant improvements in the upward movement of the hyoid bone and the amount of the UES opening during swallowing were observed [62]. In addition, the resistive jaw-opening exercise (RJOE) was postulated using elastic bands. The RJOE was effective for improving hyoid movement, reducing aspiration, and initiating oral intake in patients with dysphagia after stroke [63,64].

### 2.5. Swallow Resistance Exercise

Recently, a swallow resistance exercise device was developed. This device consists of a cotton fabric strap affixed using a VELCRO fastener at both ends for customized fitting of the device when the strap is wrapped around the neck. A concave flexible plastic disk is affixed to the middle of the strap assembly serving as a support structure for an inflatable polyethylene bag that applies an external force to the laryngeal cartilage for resisting anterior and superior deglutitive laryngeal movement. An external pressure may be applied to the thyroid by partially inflating the bag to a specific pressure reading on the gauge. This exercise resulted in a significant increase in the maximum UES opening, superior and anterior laryngeal excursion, and posterior pharyngeal wall thickness in the elderly [65].

### 2.6. Lingual Exercise

The Iowa Oral Performance Instrument (IOPI) (IOPI Medical, Woodinville, WA, USA) is a commonly used instrument for lingual exercises. The IOPI is a handheld manometry device with a 1/2 teaspoon-sized air-filled bulb, which is placed on the upper surface of the tongue. Anterior placement means that the flat front end of the bulb is positioned just behind the teeth, whereas posterior placement involves aligning the flat front end of the bulb to the anterior edge of the first molar tooth. Furthermore, this instrument provides visual biofeedback [66]. Patients with stroke and dysphagia and healthy older individuals exercised the anterior and posterior portions of the tongue one after the other by performing 10 repetitions of the exercise, three times a day on each of the three days of the week for 8 weeks. All subjects significantly increased the lingual maximal isometric pressure [67,68]. In addition, tongue strength improvements by resistance training were reported following acquired brain injury [69]. This exercise does appear to be effective in reducing thin liquid vallecular residue [66]. One study has failed to show significant improvements in tongue strength in patients with oropharyngeal cancer who underwent radiotherapy; however, the sample size in that study was small (n = 23) [70]. The resistive load for lingual exercises using the IOPI is controversial. From the analysis of healthy older adults over 70 years of age, tongue-strengthening exercises at a resistive load of one repetition maximum (1 RM) are the most effective, whereas lowering the resistive load leads to an increased success rate. After an 8 week exercise program, no de-training effects were recorded at least 4 weeks’ observation [71].

**Figure 4:**
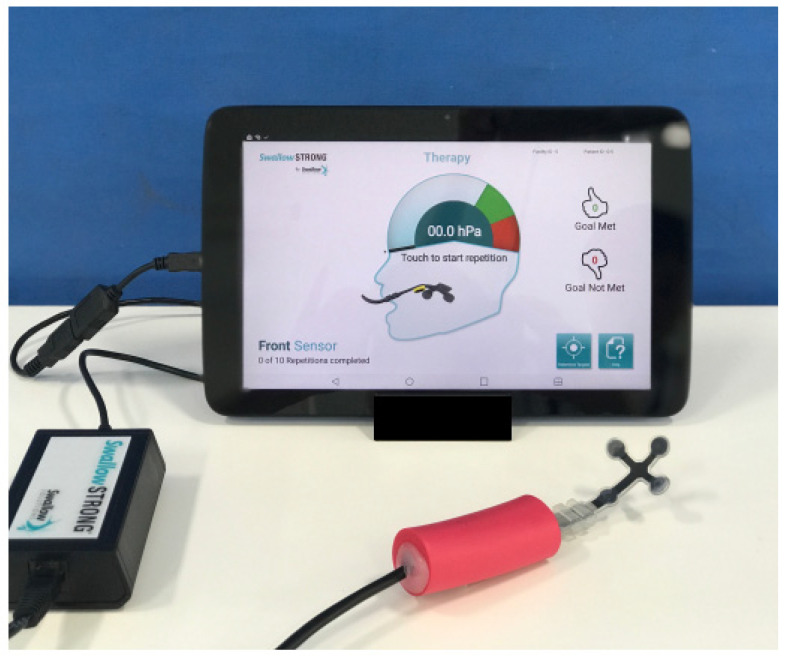
The Swallow STRengthening OropharyNGeal (Swallow STRONG). The device consists of custom-molded mouthpiece measuring tongue pressure and the tablet providing visual feedback.

In addition, other devices are used for lingual exercises. The Swallow STRengthening OropharyNGeal (Swallow STRONG) (Figure 4) postulates that an 8 week isometric progressive resistance oropharyngeal therapy consisted of pressing specific portions of the tongue against a custom-molded mouthpiece containing multiple pressure sensors. A substantial improvement in lingual muscular strength associated with improvements in swallowing-related outcomes was observed [72]. A commercially available self-exercise device (Peco-Panda, JMS, Hiroshima, Japan) is made of rubber and designed to push the semicircular training part by the anterior part of the tongue. This has five types of hardness to adjust the 60–80% strength of the maximum tongue pressure load measured using a tongue pressure measurement device. After the 8 week resistance exercise program, 30 times/set, 3 sets/day, 5 days/week, using this self-exercise device, 16 community-dwelling people aged 65 or over showed an 11.5% increase in maximum tongue pressure and 4.53 s increase in endurance of tongue pressure [73].

### 2.7. Expiratory Muscle Strength Training (EMST)

**Figure 5:**
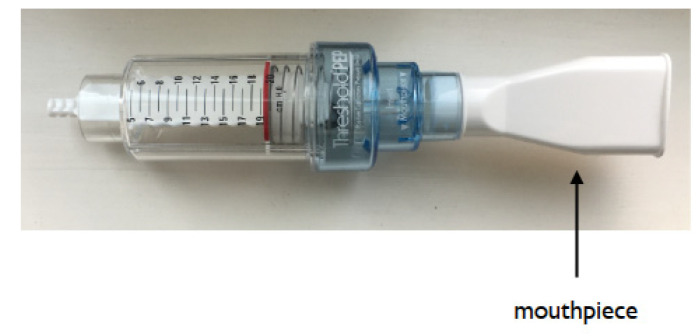
An example of EMST device (Threshold PEP)**.** The subjects are asked to open their mouth following maximum inhalation and place the mouthpiece between the lips.

The respiratory function affects deglutition. A cough peak flow (CPF) of at least 160 L/min is needed to clear airway debris, and a CPF of 270 L/min is the minimum requirement for preventing respiratory failure [74,75]. EMST is possible by using a simple device (Figure 5). Four weeks of EMST for 20 min/day, 5 days/week improved swallowing safety in patients with Parkinson’s disease [32]. Four to eight weeks of EMST increased the maximum expiratory pressure (MEP), maximum hyoid displacement, suprahyoid muscle activity, and swallowing safety in patients with amyotrophic lateral sclerosis, stroke, and head and neck cancer [76,77,78,79]. In addition, 4 months of EMST improved pulmonary and swallowing functions in patients with Huntington’s disease [80]. Usually, patients were asked to open their mouth following maximum inhalation and locate the EMST mouthpiece between the lips. They were instructed to blow strongly and fast. The pressure release valve is open when expiratory pressure exceeded the MEP set for the device. An MEP of 30–75% was commonly used for this resistance training exercise.

### 2.8. Neuromuscular Electrical Stimulation (NMES)

**Figure 6:**
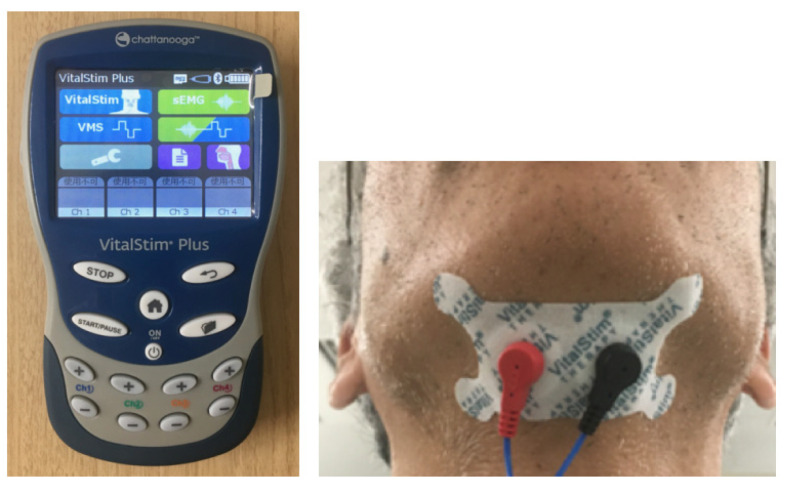
Neuromuscular electrical stimulation system (VitalStim^®^ Plus). The electrodes were attached to the submental area.

NMES is one of the most frequently recommended procedures by clinicians in the USA. for patients with dysphagia [81]. The VitalStim^Ⓡ^ Plus (Chattanooga, Dallas, TX, USA) (Figure 6) and the Ampcare effective swallowing protocol (ESPTM) (Ampcare, LLC, Fort Worth, TX, USA) are commercially available NMES products for patients with dysphagia. Different from the aforementioned procedures, NMES can be applied to patients who have difficulty in performing voluntary exercises. Usually, the electrodes were applied to either the suprahyoid or infrahyoid muscles. NMES to the digastric or thyrohyoid muscles restored the normal swallowing function in 35% of patients with severe stroke-induced dysphagia [82]. Marked improvements were reported in 20 of 23 patients with moderate-to-severe dysphagia following NMES of the thyrohyoid muscle during swallowing [83]. However, the selective stimulation of the thyrohyoid muscle by surface electrodes seemed difficult because this muscle was overlain with the sternohyoid muscle [84]. Nevertheless, NMES for the suprahyoid muscles showed positive effects in treating stroke and Parkinson’s disease [85,86]. Alternatively, NEMS for the infrahyoid muscles has been advocated as a resistance exercise against voluntary swallowing. First, NMES lowered the hyoid bone, at which condition patients were instructed to voluntarily perform deglutition [87,88]. The efficacy of NMES on patients with dysphagia has been demonstrated in meta-analyses [35,36]. Two mechanisms have been suggested to explain the training effects of NMES. The first mechanism proposes that augmentation of muscle strength using NMES occurs in a similar manner to muscle strength augmentation using voluntary exercises. This mechanism would require NMES strengthening protocols to follow standard strengthening protocols, which call for a small number of repetitions with high external loads and a high intensity of muscle contraction. The second mechanism proposes that muscle strengthening following NMES training results from a reversal of voluntary recruitment order with a selective augmentation of type II muscle fibers. Because type II fibers have a higher specific force than type I fibers, selective augmentation of type II muscle fibers will increase the overall muscle strength [89].

### 2.9. Repetitive Peripheral Magnetic Stimulation (rPMS)

**Figure 7:**
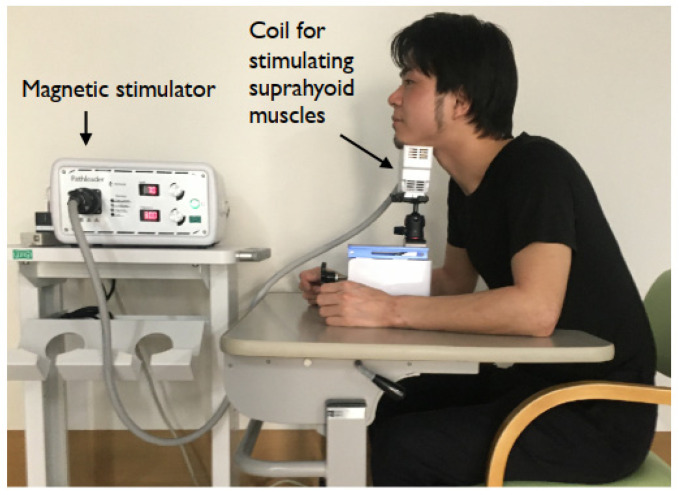
Repetitive peripheral magnetic stimulation system (Pathleader™). The smaller coil was developed to stimulate suprahyoid muscles.

Sometimes, NMES for the suprahyoid muscles causes pain and prevents strong muscle contractions. In addition, skin preparation for surface electrodes, such as shaving and wiping with alcohol in the submental area, takes time. One solution is to use rPMS for the suprahyoid muscles. rPMS does not stimulate nociceptors in the skin and can be used for high-intensity stimulation with less pain [90,91]. The coil used for rPMS does not need to be in direct contact with the skin; thus, skin preparation, including shaving, is unnecessary. One-time 10 min rPMS (1200 pulses) using MMC-90 Parabolic Coil (MagVenture Company, Farum, Denmark) improved swallowing speed and capacity in patients with stroke [92]. The disadvantage of PMS is the larger size of the coil to stimulate the suprahyoid muscles; however, the smaller coil in rPMS was recently developed. rPMS at rest elevated the hyoid bone more than NMES, and in rPMS, patients had almost the same extent of normal drinking of 10 mL liquid [90]. Two-week rPMS (5400 pulses/day) of the suprahyoid muscles significantly increased the strength of the suprahyoid muscles and had a seemingly better effect than a head-lift exercise for 2 weeks [93]. At the current version of the rPMS system (Pathleader™, IFG Corporation, Sendai, Japan), delivering 1800 pulses by 30 Hz stimulation is possible in only 2 min with an on time of 2 s and off time of 2 s, and this system seems to have good feasibility for patients with dysphagia [94] (Figure 7).

## 3. Conclusions

Resistance exercises and nutritional interventions have positive effects on the treatment of sarcopenia [95]. For sarcopenic dysphagia, resistance exercises in addition to nutritional support seems important; however, whether rehabilitation procedures are useful and which procedures are effective remains unclear. In this narrative review, we reported possible rehabilitation procedures for managing sarcopenic dysphagia. These resistance exercises are effective in treating stroke-induced dysphagia, patients with neuromuscular diseases, and heathy old people. We expect that they are also effective in treating sarcopenic dysphagia; however, no evidence exists on their effectiveness in managing sarcopenic dysphagia. The rehabilitation procedures other than resistance exercises may be effective in treating sarcopenic dysphagia. In addition, swallow resistance exercise, lingual exercise, EMST, NMES, and rPMS need devices, so they may not be applied in some institutions and/or at home. Dysphagia is a major factor preventing individuals from ingesting food and drinks orally. Further studies including randomized clinical trials should be conducted. We hope that some of these procedures or new methods will be useful in treating sarcopenic dysphagia.

## Data Availability

The data presented in this study are available on reasonable request from the corresponding author.
